# Supplementary material for: SPIN enables high throughput species identification of archaeological bone by proteomics
Source: Nat Commun. 2022 May 5;13:2458. doi: 10.1038/s41467-022-30097-x (PMC9072323; doi:10.1038/s41467-022-30097-x)
Supplement: Supplementary file 1 — Supplementary Information [file 41467_2022_30097_MOESM1_ESM.pdf]

SPIN - Species by Proteome INvestigation enables high throughput species identification of archaeological bone by proteomics

Supplementary Information

Patrick Leopold Rüther[1,*], Immanuel Mirnes Husic[1], Pernille Bangsgaard[2], Kristian Murphy Gregersen[3], Pernille Pantmann[4], Milena Carvalho[5,6], Ricardo Miguel Godinho[5], Lukas Friedl[5,7], João Cascalheira[5], Alberto John Taurozzi[2], Marie Louise Schjellerup Jørkov[8], Michael M. Benedetti[5,9], Jonathan Haws[5,10], Nuno Bicho[5], Frido Welker[2], Enrico Cappellini[2], and Jesper Velgaard Olsen[1,*]

[1]*Novo Nordisk Foundation Center for Protein Research, University of Copenhagen, Denmark*
[2]*Globe institute, University of Copenhagen, Denmark*
[3]*Institute of Conservation, Royal Danish Academy, Danmark*
[4]*Dept. of Archaeology, Museum Nordsjælland, Denmark*
[5]*Interdisciplinary Center of Archaeology and Evolution of Human Behavior, University of Algarve, Portugal*
[6]*Dept. of Anthropology, University of New Mexico, Albuquerque, USA*
[7]*Dept. of Anthropology University of West Bohemia, Czech Republic*
[8]*The Laboratory of Biological Anthropology, Dept. of Forensic Medicine, University of Copenhagen, Denmark*
[9]*Dept. of Earth and Ocean Sciences, University of North Carolina Wilmington, USA*
[10]*Dept. of Anthropology, University of Louisville, USA*
[*]*Correspondance to patrick.ruether@cpr.ku.dk; jesper.olsen@cpr.ku.dk*

Contents

Sample preparation optimization **3**

Supplementary Note 1: Reagent combinations for demineralization and extraction . . 3

Supplementary Note 2: Demineralization time 5

Supplementary Note 3: Extraction time 8

Supplementary Note 4: Reduction and alkylation 8

Supplementary Note 5: Protein aggregation capture optimization 11

Supplementary Note 6: Digestion efficiency with different sample preparation methods 13

Data acquisition optimization **14**

Supplementary Note 7: Gradient length for DDA analysis 14

Supplementary Note 8: DIA acquisition method 16

Supplementary Note 9: Spectral library acquisition optimization 19

Supplementary Note 10: Species libraries for SPIN 21

In-depth view on SPIN results **23**

Supplementary Note 11: Absolute sequence coverage 23

Supplementary Note 12: Overlap of peptide identifications 23

Supplementary Note 13: Gene-wise sequence coverage 27

Supplementary Note 14: Protein deamidation quantified by SPIN 28

Supplementary Note 15: Annotation of DIA spectra - example of a potential great ape 30

Supplementary Note 16: Phylogeny of the complete SPIN database 33

Supplementary Note 17: Timings and throughput of SPIN analysis 35

Sample preparation optimization

Supplementary Note 1: Reagent combinations for demineralization and extraction

Objectives

Combining the demineralization and protein extraction in a single step is desirable for optimizing throughput and sensitivity. It saves centrifugation and pipetting steps, which adds up to significant time and cost savings when preparing hundreds of samples. Furthermore, it can be assumed that reducing the amount of sample handling and plastic contact to a minimum benefits sensitivity. The challenges of combining the two steps are incompatibility of the reagents with each other or with the dissolved bone components, which would both impair the protein retrieval. We tested several combinations for stability of the demineralization solution, i.e. absence of insolubles falling out of solution, successful aggregation of the proteins on paramagnetic beads without precipitation or separation of the organic and aqueous phases, and the final peptide identification rates. Each experiment was conducted with 10 mg Pleistocene mammoth bone powder using the extraction agents at the final concentrations indicated in table S1.

Results

Every tested combination of the chaotropic protein solubilization agent Gnd/HCl with EDTA or acids for demineralization resulted in an unstable solution with precipitation of EDTA or Gnd/HCl (table S1). Similarly poor results were obtained with the ionic detergent SDS, which precipitated in the presence of HCl and formed insolubles during the demineralization with EDTA. Therefore, we opted for the non-ionic detergent NP-40. The combination with EDTA, hydrochloric, formic, citric, and phosphoric acid were stable, during the demineralization and extraction, but the extracted proteins could only be aggregated on paramagnetic beads, in case of HCl and EDTA. In case of acetic, formic, citric, and phosphoric acid, the beads stayed in suspension, which was

a sign for unsuccessful PAC. The three visually successful combinations EDTA + NP-40 with PAC by 70 % ACN, HCl + NP-40 with PAC by heating to 80 C, and HCl + NP-40 with PAC by 70 %ACN were subjected to on-bead digestion. Depending on the protein and mineral concentrations, PAC with organic solvent sometimes resulted in phase separation, which could be mitigated by adding 70 % ACN 30 % water until the phases mixed.

Table S1 | Combinations of demineralization and protein extraction agents. Bone powder was prepared from a Pleistocene mammoth bone fragment and 10 mg were used for testing each combination of demineralization and extraction agents. Reagent final concentrations given as percentage by volume or molarity. Stability of the combined demineralization solution and successful bead aggregation were assessed visually. Combinations without precipitation of reagents or insoluble bone minerals marked with a ″+″ in the ″Stable Combination″ column. Successful aggregation of paramagnetic beads during PAC marked with a ″+″ in the ″Bead Aggregation″ column, if the beads clumped together and the liquid phase did not separate into organic and aqueous phases. Successful protein capture as determined by peptide yield >2 µg (absorption at A_{280nm}) and > 1000 peptide identifications by LC-MS/MS (Fig. S1) is marked with ″+″ in the ″Protein Capture″ column.

Demineralization Agent	Extraction Agent	Protein Aggregation Method	Stable Combination	Bead Aggregation	Protein Capture
EDTA (0.5 M)	Guanidine- HCl (3 M)	Heat (80 C)			
EDTA (0.5 M)	Guanidine- HCl (3 M)	10% TCA			
EDTA (0.5 M)	Guanidine- HCl (3 M)	2.6 M Ammonium Sulfate			
EDTA (0.5 M)	Guanidine- HCl (3 M)	70% Acetonitrile			
EDTA (0.5 M)	SDS (1-5 %)	-			
EDTA (0.5 M)	NP-40 (0.1-1%)	70% Acetonitrile	+	+	
Acetic Acid (5-30 %)	SDS (1-5 %)	-			
Formic Acid (5-30 %)	NP-40 (0.1-1 %)	Acetonitrile (60 - 80 %)	+		
Citric Acid (5-30 %)	NP-40 (0.1-1 %)	Acetonitrile (60 - 80 %)	+		
Phosphoric Acid (5-30 %)	NP-40 (0.1-1 %)	Acetonitrile (60 - 80 %)	+		
HCl (5 %)	SDS (1-5 %)	-			
HCl (5 %)	Guanidine- HCl (3 M)	-			
HCl (5 %)	NP-40 (1%)	Heat (80 C)	+	+	
HCl (5 %)	NP-40 (1%)	Acetonitrile (60 - 80 %)	+	+	+

The peptides were desalted by C18 StageTips and analyzed spectrophotometrically at A_{280nm} and by LC-MS/MS resulting in over five times more peptide identifications in case of HCl demineralization compared to EDTA demineralization (fig. S1). Most of the peptides identified in the EDTA-demineralized sample were also detected in the HCl experiment. Spectrophotometrical peptide yields (A_{280nm}) were used for rough estimation of peptide recoveries and adjusting the LC-MS/MS injection amounts. However, the results were very unreliable due to the low amounts of aromatic amino acids in bone proteins and are therefore not included, in the study. The combination of HCl demineralization with NP-40 extraction and aggregation by heat was excluded, be-

cause the spectrophotometrical analysis suggested that no peptides were present. The deamidation rate was higher after HCl demineralization, which is probably caused by the acid but might be partially due to the increased proteome depth. Focussing on the genes with the highest peptide identification rates, the sequence coverage was significantly lower with EDTA for most genes. However, similar or slightly higher sequence coverage was achieved with EDTA, in case of the two most abundant genes COL1A1 and COL1A2.

Conclusions

The only combination of a demineralization and extraction agent that was compatible with PAC and resulted in good peptide recovery was HCl with NP-40 followed by aggregation with ACN. Although the deamidation rates were elevated, the number of peptide identifications and therefore the protein sequence coverage were greatly increased. We moved on to further optimization of this sample preparation procedure. For applications with focus on protein modifications like deamidation, particularly in the highly abundant collagen type one chains, an EDTA-based workflow could be a beneficial due to its lower impact on protein integrity.

Supplementary Note 2: Demineralization time

Objectives

Longer demineralization has the potential to improve the release of proteins from the hydroxyapatite matrix. However, it comes at the risk of increased deamidation rates and unspecific protein hydrolysis. We tested an extension of the overnight demineralization time to two and three days.

Results

An extension of the demineralization time beyond one day reduced the number of identified peptides (fig. S2). The deamidation rate stayed within a range of 49 - 55 % with the highest rate reached after 2 days of bone demineralization. While the

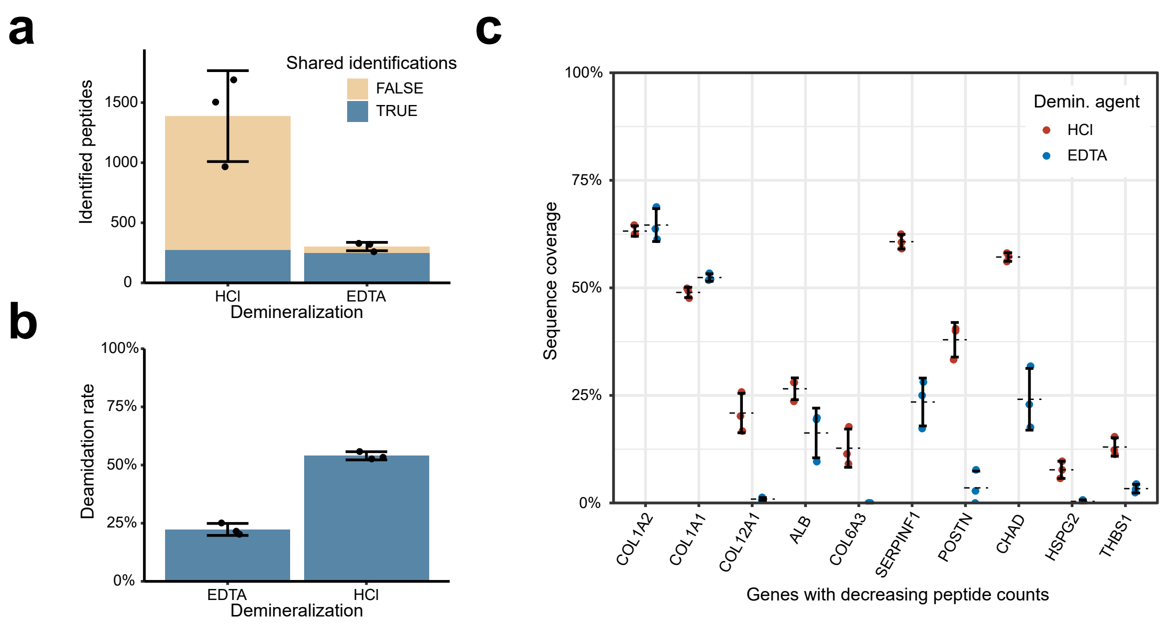

Fig. S1 | Combined bone demineralization/extraction optimization. Ten mg bone chips from a medieval sheep were processed by combined demineralization and extraction using either HCl or EDTA as demineralization agents in combination with NP-40 for protein solubilization. Proteins were cleaned up and digested by PAC followed by LC-MS/MS analysis on a 21 min gradient (60 samples/day) and DDA acquisition. **a**, Number of peptide identifications obtained with the two different demineralization agents. Bars represent the mean of n = 3 technical replica experiments starting from the same batch of bone powder with error bars centered around the mean total peptide identifications indicating the standard deviation. Peptide identifications shared by both methods are shown in dark blue and identifications only observed with one demineralization agent are shown in sand color. **b**, Deamidation rates obtained with the different demineralization agents and calculated as relative precursor counts. Bars represent the mean deamidation rate of n = 3 technical replica experiments starting from the same batch of bone powder with error bars centered at the mean indicating the standard deviation. **c**, Sequence coverage of the top ten genes with most peptide-spectrum-matches displayed with decreasing order of total number of PSMs. Red datapoints indicate demineralization with HCl and blue datapoints with EDTA. Dashed lines represent mean sequence coverage in n = 3 technical replica experiments starting from the same batch of bone powder and error bars indicate standard error centered around the mean.

absolute number of identified peptides decreased for any type of cleavage specificity, we observed that the tryptic population decreased most.

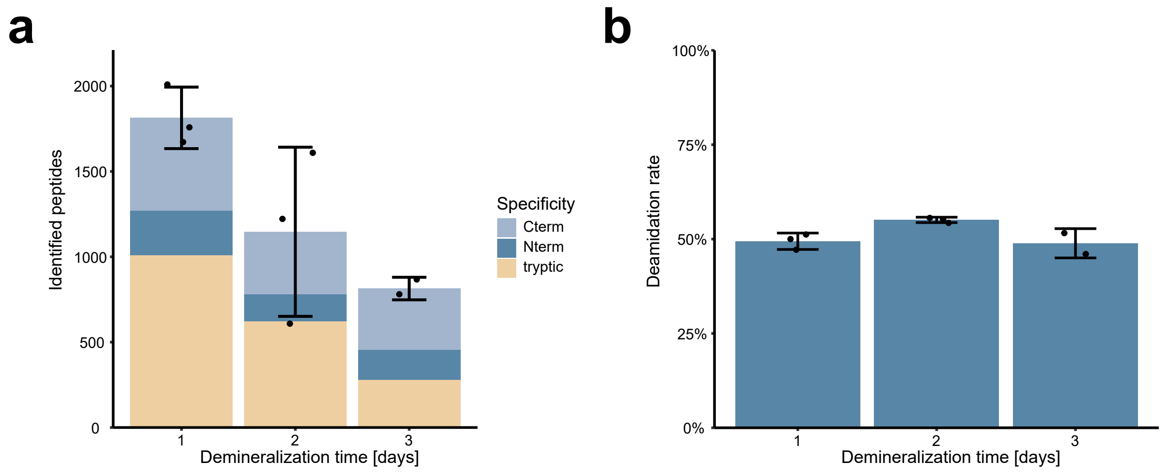

Fig. S2 | Demineralization time optimization. Ten mg bone chips from a medieval sheep were processed by combined HCl demineralization at room temperature and NP-40 extraction with a demineralization time of one (16 h, overnight), two, or three days. Proteins were cleaned up by PAC using 70 % ACN for aggregation followed by LC-MS/MS analysis on a 21 min gradient (60 samples/day) and DDA acquisition. **a**, Number of peptide identifications obtained with different demineralization times. Bars represent the mean peptide identifications in n = 3 (n = 2 for three days) technical replica experiments starting from the same batch of bone chips separated by enzyme specificity shown by color with sand color for tryptic, dark blue for semi-tryptic with non-tryptic C-terminus and light blue for semi-tryptic with non-tryptic N-terminus. Error bars centered around the mean overall peptide identifications indicate the standard deviation of the total number of identified peptides. **b**, Mean deamidation rates calculated as relative precursor counts and error bars centered around the mean indicating the standard deviation in the n = 3 (n = 2 for three days) technical replica experiments starting from the same batch of bone chips.

Conclusions

Contrary to our expectations, longer demineralization resulted in fewer peptide identifications. This was likely due to an increase in protein modification and unspecific hydrolysis in the acidic demineralization/extraction solution. Another reason could be an unproportionally high release of collagen, which "masks" other proteins during LC-MS/MS analysis due to the dynamic range limits. For the SPIN protocol, we therefore opted for the short overnight demineralization. We decided against shortening the demineralization time to less than 16 h because this would make the workflow impractical, particularly for routine analysis. An alternative approach to less damag-

ing demineralization could be reducing the temperature, which will be tested, in the future.

Supplementary Note 3: Extraction time

Objectives

Protein extraction required elevated temperatures to gelatinize the collagens. Longer extraction times could improve protein solubilization, but the acidic demineralization/extraction solution can potentially facilitate protein hydrolysis and deamidation, at these temperatures. We compared protein extraction at one and two hours.

Results

One hour of protein extraction resulted in significantly higher peptide identifications (fig. S3). The deamidation rate only varied marginally between the two time points.

Conclusions

We selected an extraction time of one hour for the SPIN workflow. The low increase of deamidation with two-fold longer protein extraction at 80 °C was counter intuitive but the number could be affected by the low identification rates. Similar to the extended demineralization time, prolonged protein extraction could lead to disproportionately high release of collagen, which has the risk of suppressing other protein identifications.

Supplementary Note 4: Reduction and alkylation

Objectives

Reduction and alkylation of cysteine residues plays an important role in protocols for bottom-up proteomics because disulfide bonds would otherwise impair protein digestion and identification of cysteine-containing peptides. However, only few

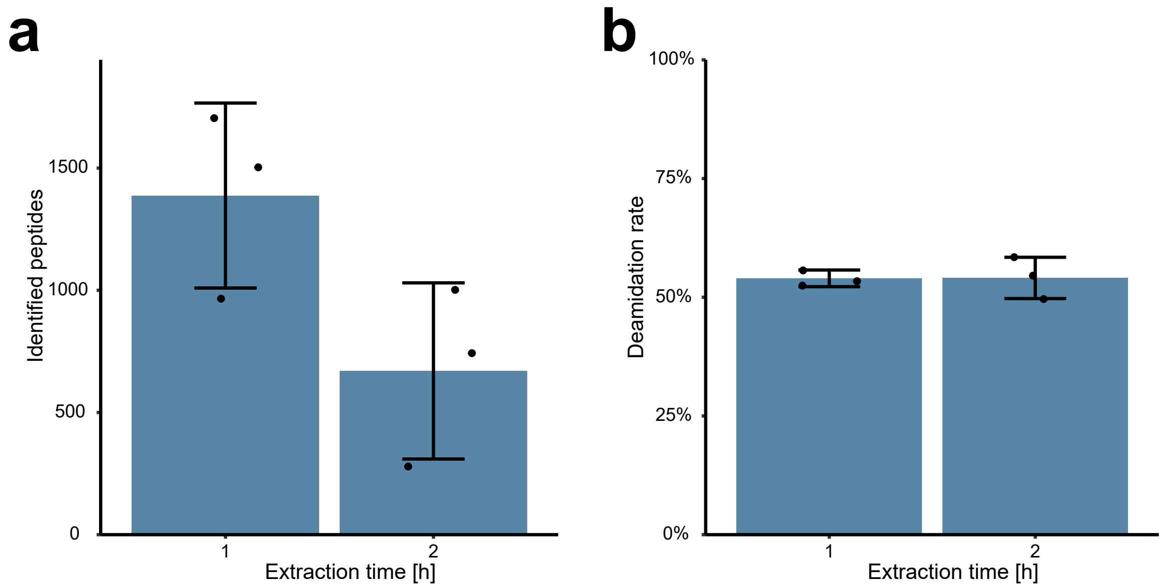

Fig. S3 | Extraction time optimization. Ten mg bone chips from a medieval sheep were processed by combined HCl demineralization at room temperature and NP-40 extraction with protein extraction at 80 °C for one or two hours. Proteins were cleaned up by PAC using 70 % ACN for aggregation followed by LC-MS/MS analysis on a 21 min gradient (60 samples/day) and DDA acquisition. **a**, Number of peptide identifications obtained with different extraction times. Each bar represents the mean peptide identifications in n = 3 technical replica experiments starting from the same batch of bone chips. Error bars centered at the mean show the standard deviation. **b**, Mean deamidation rates calculated as relative precursor counts and error bars indicating the standard deviation in three replica experiments.

cysteine-containing peptides are traditionally identified in palaeoproteomics studies, because the highly-reactive thiols and even the less reactive disulfide bridges can be oxidized or otherwise modified during protein diagenesis. This raises the question, whether spending time and resources on these extra steps is necessary. In case of the SPIN protocol, the protein extract has a low pH, which excludes the use of chloroacetamide or iodoacetamide for alkylation. We therefore opted for a combination of TCEP and NEM, which are theoretically able to work at a lower pH.

Results

Only few cysteine-containing peptides could be identified, in the experiments. Nevertheless, we observed that more peptides with the NEM modification were identified

with the included reduction and alkylation step. It is likely that the identifications of peptides with unmodified cysteine residues and the single identification of a peptide modified with NEM in the samples without reduction and alkylation were false positives. While the additional identifications of cysteine-containing peptides were only few, the reduction and alkylation step had a positive effect on the overall peptide identifications.

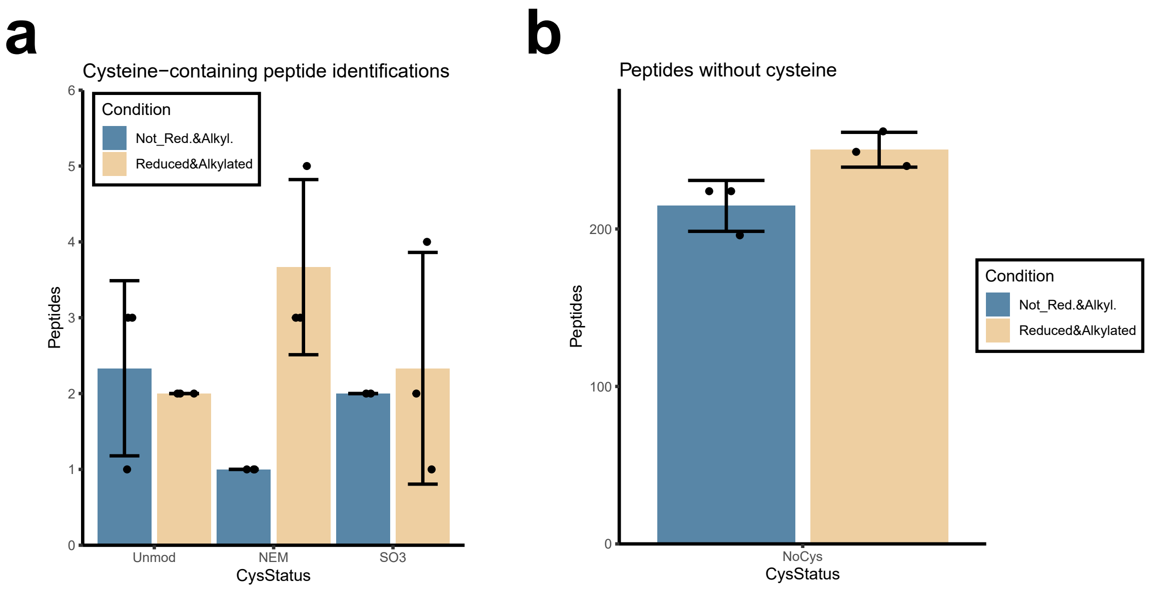

Fig. S4 | Cysteine reduction and alkylation. Ten mg bone chips from a medieval sheep were processed by combined HCl demineralization at room temperature, overnight. Before the extraction step, 10 mM TCEP and 20 mM NEM were added or not added to the solution. The subsequent extraction was carried out with standard settings at 80 °C for 1 h. Proteins were cleaned up by PAC using 70 % ACN for aggregation followed by LC-MS/MS analysis on a 21 min gradient (60 samples/day) and DDA acquisition. **a**, Cysteine-containing peptide identifications in three different states: Unmodified thiol, alkylated with NEM, and trioxidized to sulfonic acid. Bars indicate the mean of n = 3 technical replicate experiments starting from the same batch of bone chips. Error bars centered at the mean indicate standard deviation. Reduction and alkylation is indicated in dark blue for experiments without reduction and alkylation and sand color for experiments with reduction and alkylation. **b**, Same plot as in (a) for peptides without Cysteine residues.

Conclusions

With reduction and alkylation, we were able to identify more peptides with cysteine residues. However, the ten additional peptide identifications with cysteines did not justify the added effort, by themselves. The unexpected positive effect was the increased identification rate of peptides without cysteine residues. Possible explanations are an improvement in digestion efficiency due to reduction of disulfides or other compounds in the PAC digestion mixture. We therefore decided to include the reduction and alkylation step with TCEP and NEM in the SPIN protocol.

Supplementary Note 5: Protein aggregation capture optimization

Objectives

The applicability of the PAC method [1, 2] for the generation of peptides from archaeological bone was already demonstrated by Cleland et al. but not yet optimized for this specific sample type [3]. Ancient bone protein extracts differ from a classic tissue or cell line lysate, both in the proteinaceous and non-proteinaceous content. These have an impact on the efficiency of PAC. The high concentration of solubilized salts from the bone demineralization tend to fall out of solution or cause a separation of the aqueous phase when mixed with organic solvents. The proteins, on the other hand, have been subjected to degradation and protein hydrolysis into shorter fragments. While the high salt concentrations require the use of more polar organic solvents like ethanol or lower final concentrations of organic solvents, the shorter protein fragments require the opposite for effective aggregation. We therefore tested the less polar solvent acetonitrile and the more polar ethanol, each at five different final concentrations during PAC.

Results

Based on peptide identifications, acetonitrile outperformed ethanol at all tested solvent concentrations. While the highest number of peptide identifications could be reached at a concentration of 70 % ACN. However, this required a relatively large di-

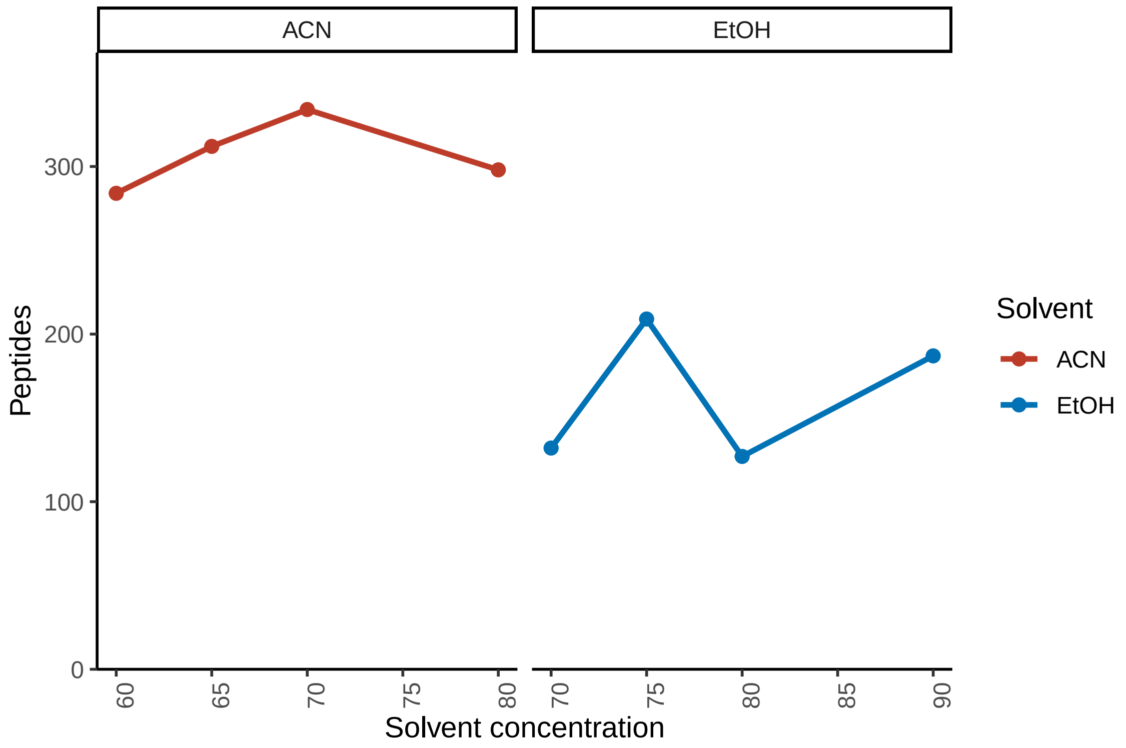

Fig. S5 | PAC optimization. Ten mg bone chips from a medieval sheep were processed by the standard combined demineralization/extraction protocol of the SPIN workflow. The subsequent protein aggregation capture was facilitated with acetonitrile (ACN) at 60, 65, 70, 75, or 80 % or with ethanol (EtOH) at 70, 75, 80, 85, or 90 % final concentration. Peptides were analyzed with by LC-MS/MS on a 21 min gradient (60 samples/day) and DDA acquisition. Each datapoint represents the number of identified peptide sequences in a single experiment. Red indicates usage of ACN and blue EtOH as the aggregation solvent.

lution of the input solution to prevent phase separation, which was less severe at 60 % ACN.

Conclusions

Our results indicated that ACN was the superior solvent for PAC of ancient proteins from a combined demineralization/extraction procedure. The higher 70 % concentration lead to phase separation more often, which required tedious dilution with 70 % ACN in water, which was not ideal for scale-up. We, therefore, decided to use 60 % ACN in the final SPIN workflow due to the better robustness, although slightly higher

peptide identifications could be achieved with 70 % ACN.

Supplementary Note 6: Digestion efficiency with different sample preparation methods

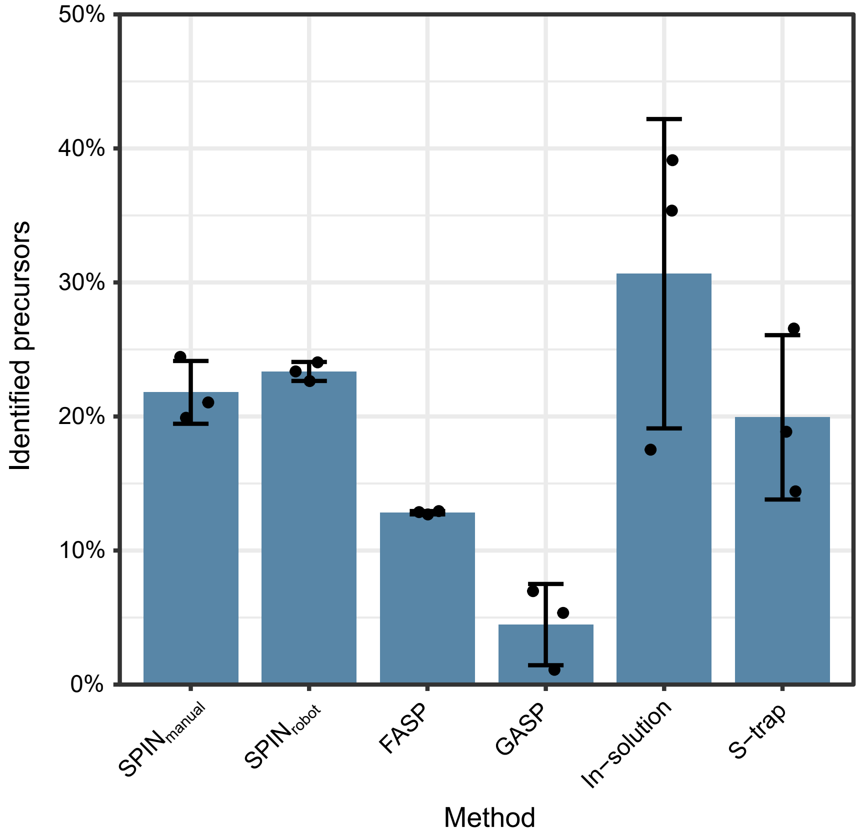

Fig. S6 | Digestion efficiency of different sample preparation methods. Missed cleavage results are based on the data generated for Fig. 1b. Bars represent mean missed cleavage rates calculated as number of PSMs containing a non-C-terminal K or R residue divided by the total number of PSMs for n = 3 technical replicate experiments starting from the same batch of bone powder. Error bars are centered at the mean and indicate standard deviation.

Objectives

In addition to peptide identifications and tryptic cleavage specificity, we wanted to assess the digestion efficiency for the different tested sample preparation methods.

As a proxy, we utilized the number ofidentified precursors with and without internal tryptic cleavage sites, i.e. ‘missed cleavages‘.

Results

We observed very similar missed cleavage rates between 20 - 23 % for the sample preparation following the PAC and S-trap workflows. The digestion efficiency was lowest in the samples prepared with the in-solution method and highest for FASP and GASP (Fig. S6).

Conclusions

The three aggregation-based sample preparation methods expectedly resulted in similar digestion efficiencies. The in-solution method probably performed worse due to the presence of the chaotropic agent Gnd/HCl in the solution, whereas FASP and GASP had very few missed cleavages due to the efficient buffer exchange.

Data acquisition optimization

Supplementary Note 7: Gradient length for DDA analysis

Objectives

Contrary to proteomics studies focussed on the highest possible proteome depth, the SPIN workflow was aimed at throughput and efficiency. To find the fastest possible acquisition strategy that still delivered sufficient sequence coverage for species identification, we compared four short gradients on the Evosep One LC.

Results

The shortest 5.6 min gradient only resulted in about 200 peptide identifications by DDA analysis (fig. S7), which translated to low sequence coverage and was not ideal for consistent species assignmen. When increased to 11 min,

the peptide identifications were more than doubled to about 450. With the two times slower 21 min gradient, only a further gain of 100 peptides could be achieved, while the 45 min gradient did not provide any further identifications compared to 21 min. The number of identified spectra per second gave an idea of the maximum identification speed over the retention time. The two shorter gradients (200 spd and 100 spd) reached over 5 identified MS2 spectra per second, while the two other gradients stayed below 3.

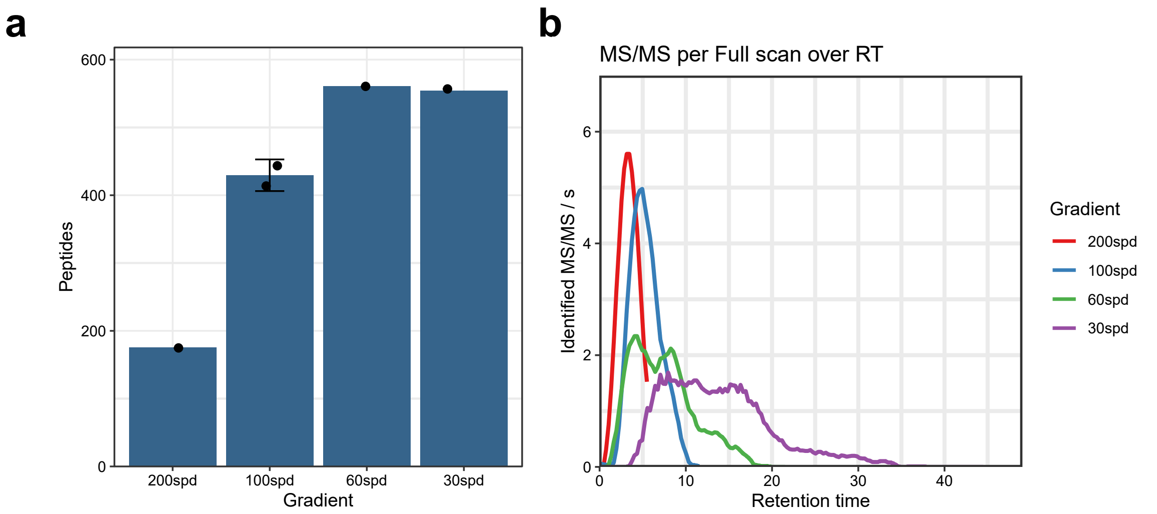

Fig. S7 | DDA gradient optimization. Ten mg bone powder from a medieval sheep were processed by the standard SPIN sample preparation workflow. Peptides were analyzed by LC-MS/MS in DDA mode on four different Evosep One gradients using the same 10 cm column: 200 samples/day (5.6 min), 100 spd (11 min), 60 spd (21 min), and 30 spd (45 min). **a**, Each bar represents the number of identified tryptic peptide sequences in a single experiment and n = 2 technical replicate experiments for the 100 spd gradient starting from the same peptide solution. The error bar for the 100 spd gradient indicate the standard deviation. **b**, The number of MS/MS spectra identified per second across the retention time in minutes for one representative experiment of the 4 different gradients. Gradient length is indicated by color with red for 200 spd, blue for 100 spd, green for 60 spd, and purple for 30 spd.

Conclusions

In the gradient comparison, the point of diminishing returns was reached at the 100 or 60 samples/day gradient. The 30 samples/day gradient would require higher loads

or potentially a longer column yielding sharper peaks to provide a benefit. The best compromise between speed and proteome coverage could be achieved with the 100 samples/day method and it was therefore included in the SPIN protocol. An added benefit of shorter gradients was the increased sensitivity due to the sharp peak shape, which resulted in higher peak intensity and therefore better MS2 signal-to-noise ration. Practically, this meant that more fragment scans could be identified per second (fig. S7b).

Supplementary Note 8: DIA acquisition method

Objectives

For the high-throughput SPIN workflow, our goal was the identification of as many peptides as possible in the smallest possible amount of acquisition time. We optimized the DIA parameters for two different gradients, 200 samples/day (5.6 min) and the 100 samples/day (11 min). The challenge of these short LC gradients is the sharp peak shape with full-width half-maximum peak widths well below 10 seconds. Depending on the MS resolution and the number of windows for precursor isolation, the DIA cycle time can be a few seconds long. This would result in very few data points per peak, which is particularly bad for quantification but also affects identification. As species identification is not heavily dependent on quantitative precision, we were able to accept longer cycle times and fewer data points per peak than a ”normal” DIA-based proteomics experiment. We, therefore, opted for a slower but more sensitive MS2 resolution of 30,000 and optimized the number of DIA windows and the overall MS2 isolation range by varying the window width. To preserve high mass identifications without losing too much time, we added three significantly wider DIA isolation windows at the high m/z end of the overall MS2 range (770 - 970 m/z). Within the lower mass range, four different window counts were compared with the lowest window count only tested with the short and the highest count only tested with the long gradient. The resulting window widths can be found in table S2 and the complete MS methods are available in the uploaded raw data.

Table S2 | DIA method optimization. All tested combinations of gradient length, DIA MS2 selection range, and window width. Values separated by a forward slash character refer to the narrow (range 1) and broad (range 2) DIA window range. Cycle times and median peak width EXT have been calculated based on the Spectronaut output and represent means of three replicates.

Gradient length [min]	MS2 (range 1/range 2) [Th]	No. of Windows (range 1/range 2)	Window width (range 1/range2) [Th]	Cycle time [s]	Median Peak Width EXT [s]
5	350-770/770-970	10/3	43/66	1.36	3.92
5	440-770/770-970	10/3	34/66	1.43	4.24
5	350-770/770-970	15/3	29/66	1.78	4.69
5	440-770/770-970	15/3	23/66	1.71	4.58
5	350-770/770-970	20/3	23/66	2.20	5.39
5	440-770/770-970	20/3	18/66	2.13	5.43
11	350-770/770-970	15/3	29/66	1.78	6.10
11	440-770/770-970	15/3	23/66	1.78	6.20
11	350-770/770-970	20/3	23/66	2.21	6.70
11	440-770/770-970	20/3	18/66	2.13	6.71
11	350-770/770-970	25/3	18/66	2.56	7.19
11	440-770/770-970	25/3	14/66	2.62	7.58

Results

The faster 200 samples/day gradient resulted in the identification of about 500 peptides, whereas 700 - 800 peptides could be identified with the slower 100 samples/day method (fig. S8a). This puts the library-based DIA results achieved with the fast 200 samples/day method in a similar or even slightly better performance category as DDA analysis using the slower 100 samples/day gradient (fig. S7a). Changing the MS2 isolation range at the low m/z end between 350 - 980 m/z and 440 - 980 m/z had no impact on the peptide and protein identifications. Similarly, changing the number of DIA windows only had minor impact on peptide and protein identifications. In case of the faster 200 samples/day gradient, the DIA method using an MS2 range of 350 - 980 m/z and 15 windows seemed slightly superior, but the difference was below statistical significance. The sequence coverage comparison indicated that the performance of all 12 DIA methods was highly similar for the highly-abundant collagen type 1 proteins (fig. S8b). However, higher collagen sequence coverage was consistently obtained with the broader MS2 isolation range of 350 - 980 m/z for both LC gradients. The coverage of the less abundant proteins was lower, in case of the 200 samples/day gradient. The sequence coverage variations between the different numbers of DIA windows were minimal.

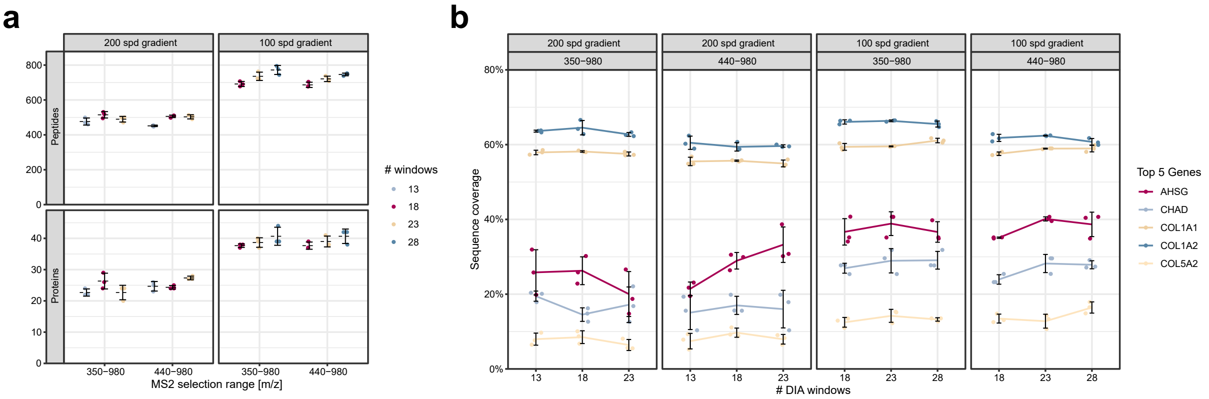

Fig. S8 | Optimization of DIA methods. Peptides were generated from sheep bone powder following the SPIN protocol. LC-MS/MS analysis was carried out using the 200 samples/day (5.6 min) and the 100 samples/day (11 min) gradients, two different isolation ranges for data-independent peptide fragmentation, and three different window width settings that translate into different DIA cycle times. For every method, the three DIA windows with the highest m/z were about 70 m/z broader compared to the other windows. **a**, Semi-specific peptide and protein identifications. Dashed lines indicate the mean number of identifications in $n = 3$ technical replicate experiments starting from the same peptide solution. Error bars centered at the mean indicate standard deviation. Colors show the number of DIA isolation windows ranging from 13 (light blue), which was only tested with the faster 200 spd gradient, over 18 (pink) and 23 (sand color), to 28 (dark blue), which was only used for the slower 100 spd gradient. **b**, Sequence coverage of the five genes with most identified peptides for each of the DIA methods. Lines indicate the mean of $n = 3$ technical replicate experiments starting from the same peptide solution and error bars represent standard deviation. Color indicates the respective gene, as indicated in the legend.

Conclusions

In the interest of higher throughput and due to the very similar numbers of peptide identifications and collagen sequence coverage between the gradients, we selected the 200 samples/day gradient for the SPIN workflow. However, it should be noted that higher sequence coverage can be obtained with the slower 100 samples/day gradient, particularly for non-collagenous proteins. We opted for the broader MS2 isolation range of 350 - 980 m/z, as it provided better collagen sequence coverage. As the number of DIA windows did not greatly affect the identification rates and sequence coverage, we decided to use 18 windows in the final SPIN protocol due to the slightly higher peptide identification rate.

Supplementary Note 9: Spectral library acquisition optimization

Objectives

The most common way of identifying peptides in DIA data is using spectral libraries, which are usually generated by offline pre-fractionation and DDA measurement of a reference sample. In case of species identification, peptides unique for a species can only be identified by library-based DIA, if a spectral library is available for that species. To make the acquisition process for a larger set of species libraries efficient, we tested four different high-pH reversed-phase offline fractionation strategies resulting in 12 or 24 fractions, which were collected without or with concatenation of 24 or 48 individual fractions, respectively. All fractions were measured using the 200 samples/day gradient to enable the best possible retention time matching between the DIA and DDA data.

Results

As expected, more modification-specific peptides and proteins were identified with 24 fractions compared to 12 (fig. S9a). The impact of concatenation on library depth was less consistent. While the numbers were slightly lower with concatenation for 12 offline fractions, they were higher, in case of 24 fractions. In theory, concatenation has the benefit of spreading the peptides more evenly over the fractions, but we hypothesize that these benefits could be outweighed by the high abundance of collagenous peptides in bone samples that tend to spread over multiple fractions. When using the four different spectral libraries for analyzing nine sheep bone samples measured by DIA, the differences in peptide identifications were much smaller (fig. S9b). Here, the number of identified peptides was only marginally higher for the library based on 24 fractions with concatenation, while both the 12- and 24-fraction libraries had slightly lower peptide identification numbers.

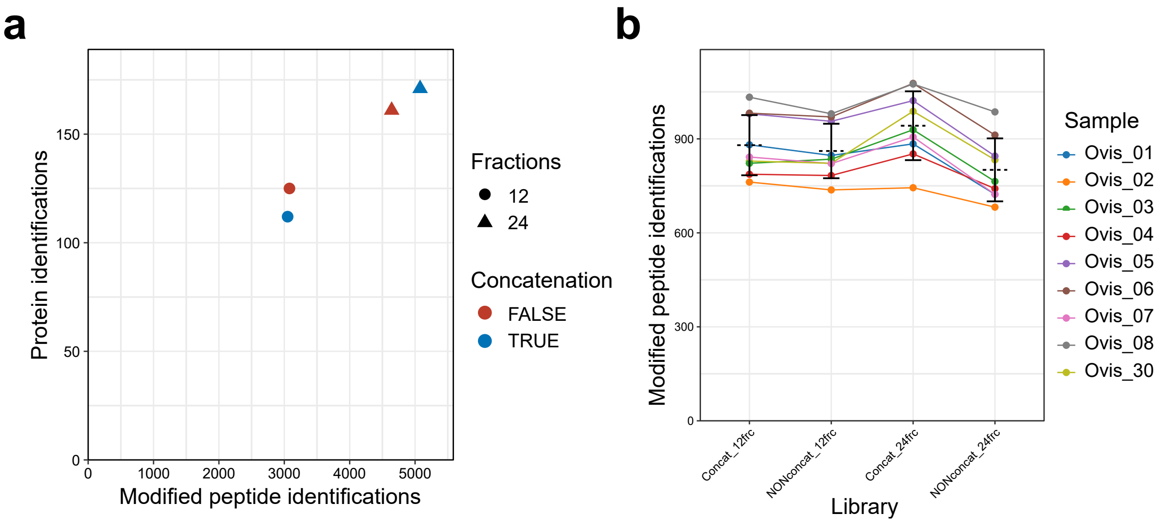

Fig. S9 | Spectral library optimization. Spectral libraries were generated from peptides generated by the SPIN sample preparation procedure with sheep bone powder. Peptides were offline fractionated into 12 or 24 fractions using high-pH reversed-phase chromatography and the 77 min gradient described in the online methods. Fractions were either collected directly or by concatenation of 24 to 12 or 48 to 24 fractions. An equivalent of 250 ng peptides were analyzed by LC-MS/MS in DDA mode using the 200 samples/day gradient. **a**, Peptide and protein identification results based on database search of the spectral library data with Maxquant using the complete sheep protein database from Uniprot. Each point represents the collective modified peptide (x-axis) or protein (y-axis) identifications for a single experiment with 12 or 24 fractions. Color indicates whether the fraction scheme included concatenation (blue) did not include concatenation (red) and shape indicates the number of offline fractions. **b**, Samples from medieval reference bones from $n = 9$ biological replicates, i.e. different sheeps, were prepared using the SPIN workflow and analyzed by the fast 200 samples/day gradient and DIA. Library-based peptide identification was carried out in Spectronaut using the four different spectral libraries. The library is displayed on the x-axis and the mean number of identified modification-specific peptide sequences is shown on the y-axis. Error bars are centered at the mean and indicate standard deviation. Different samples are highlighted by colors, as shown in the figure legend.

Conclusions

Based on the DDA results, the higher number of 24 fractions seemed clearly superior to the 12 fraction approach. However, these results did not translate into the DIA analysis results, where the type of library only had a small impact on peptide identifications. This was likely the case because of the rather short gradients used for DIA analysis complicating the identification of peptides with low abundance. As this basi-

cally canceled out the advantage of 24 offline fractions, we opted for only 12 fractions in the final SPIN protocol, as this saved library acquisition time and costs. Although the concatenated 12-fraction library resulted in slightly higher numbers of peptide identifications compared to the non-concatenated version, we chose to continue without concatenation, since the marginal increase did not justify the added complexity and lower robustness of concatenation.

Supplementary Note 10: Species libraries for SPIN

Objectives

Our proof-of-concept study of the SPIN workflow was focused on common domestic mammalian species and their wild ancestors, which could be found in Danish bone assemblages, and a few great ape species to prepare for the possibility of identifying human remains in the Portuguese sites with Neanderthal occupation. We therefore acquired a collection of reference bones with certain species origins covering 13 different large mammalian species. To ensure consistent library quality, we compared the proteome depth based on DDA analysis of the samples fractionated for library generation.

Results

Similar coverage was achieved for most species libraries in our study with the human library reaching the lowest and the goat library reaching the highest depth (fig. S10). All libraries contained at least 1000 unique peptide identifications.

Conclusions

As also shown in the main article, all libraries were sufficient for confident species assignments by SPIN (fig. 3) despite the variations in library depth. It is fair to assume that the core peptide identifications shared by all libraries was the most relevant for identifying peptides in the 200 samples/day DIA experiments. Therefore, the additional peptides in the deeper libraries might not play an important role for the purpose of high-throughput species identification Interestingly, the lowest identification rate

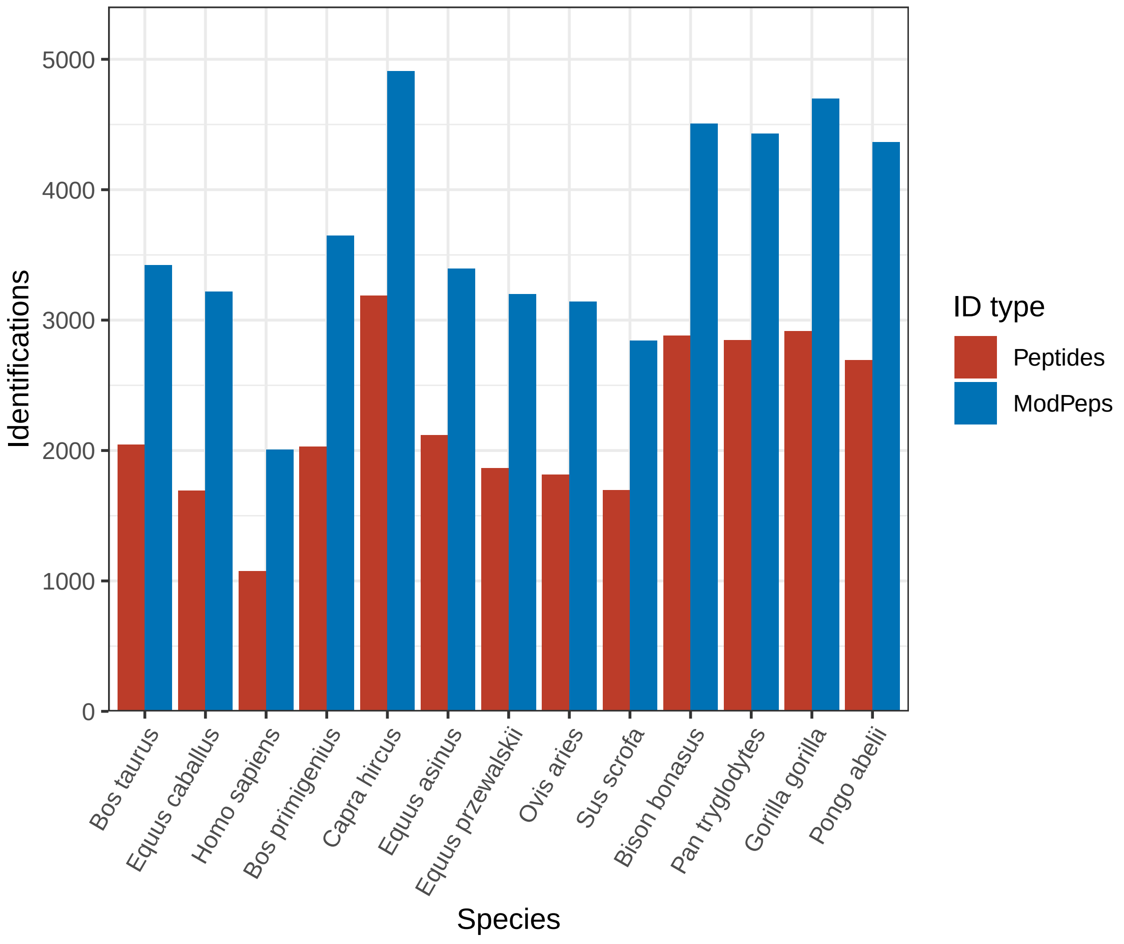

Fig. S10 | Species library depth. Spectral libraries were generated by offline fractionation into 12 fractions and DDA acquisition using the 200 spd gradient, for 13 large mammalian species. One spectral library was acquired per species. Identified peptides in each library are shown in red and identified modification-specific peptide identifications in blue.

was achieved with the most well-annotated species, human. Due to ethical sampling requirements, we did not acquire another human specimen or repeat the human library acquisition. Besides the preservation conditions of the reference samples, the database annotation state probably also played a role in the final library depth. The database could affect the library identification in both directions: Up, if a database contains more important bone protein sequences of down, if a database is very large resulting in a more conservative FDR control.

In-depth view on SPIN results

Supplementary Note 11: Absolute sequence coverage

Objectives

To assess the effective performance of the three peptide identification methods DDA, library-based DIA, and Direct DIA, we looked at the sequence coverage measured in the reference bone samples.

Results

The highest absolute coverage was achieved with library-based DIA followed by DDA and then DirectDIA (fig. S11). The variability between samples was similar between the three different data types.

Conclusions

Despite DirectDIA reaching slightly higher precursor identification rates than DDA (fig. 1c), the DDA data covered more sites indicating higher redundancy of different cleavage or modification forms within the DirectDIA results. Library-based DIA still achieved the highest absolute sequence coverage. However, the difference to DDA and DirectDIA, which was about two-fold on precursor level, went down to an advantage of about 20 % more identified sites by library-based DIA.

Supplementary Note 12: Overlap of peptide identifications

Objectives

As we measured the complete sample set in the SPIN study with two different acquisition methods (DDA and DIA) and interpreted the spectra with three different approaches (Maxquant DDA, Spectronaur library-based and DirectDIA), we wanted to check the peptide identification rates and overlaps between the three different peptide identification strategies.

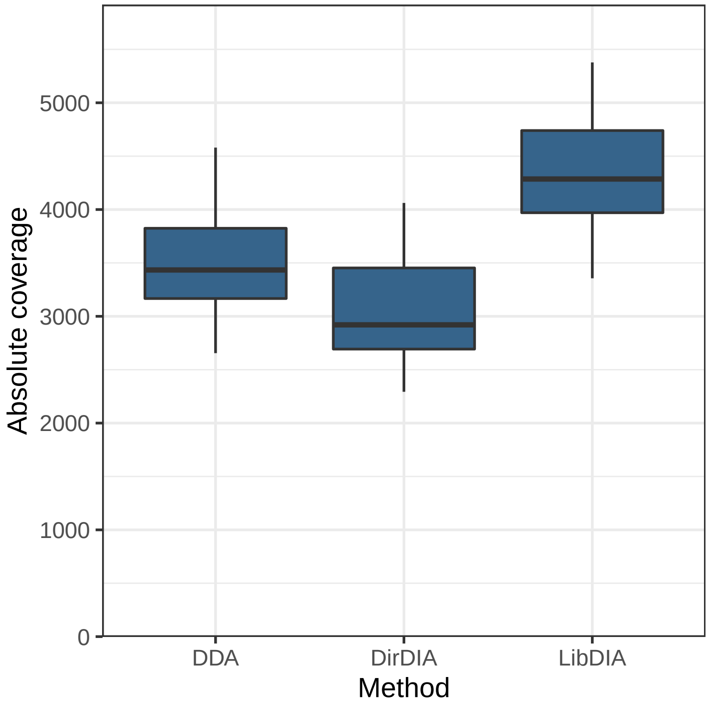

Fig. S11 | Absolute sequence coverage comparison by data type. Boxplots displaying the absolute sequence coverage obtained with the reference bone samples (n = 35 biologically independent samples) by DDA, library-based DIA "LibDIA", and Direct DIA "DirDIA". Median is indicated by a horizontal line, the lower and upper quartile by the boarders of the box, and the 1.5 x interquartile range by the whiskers.

Results

The relative distribution of peptide sequences between the three peptide identification methods was similar for all reference species (fig. S12). Despite being based on the same raw files, the relative overlap between the two different DIA search algorithm was not significantly larger than the overlap between DDA and library-based DIA. Between 9 and 14 % of the identified peptides were shared between all three methods.

Conclusions

Although we expected a larger overlap between the three methods, the species determined with each of them were mostly in agreement (fig. 3). The peptides that were only identified with one or two of the methods could be used for complementing the

results by combining multiple search engines or data acquisition methods. Based on the relatively similar distributions of identified peptides across the data sets for all the reference species, we concluded that there is little bias for any of the species, which could have been the case due to varying database and library size.

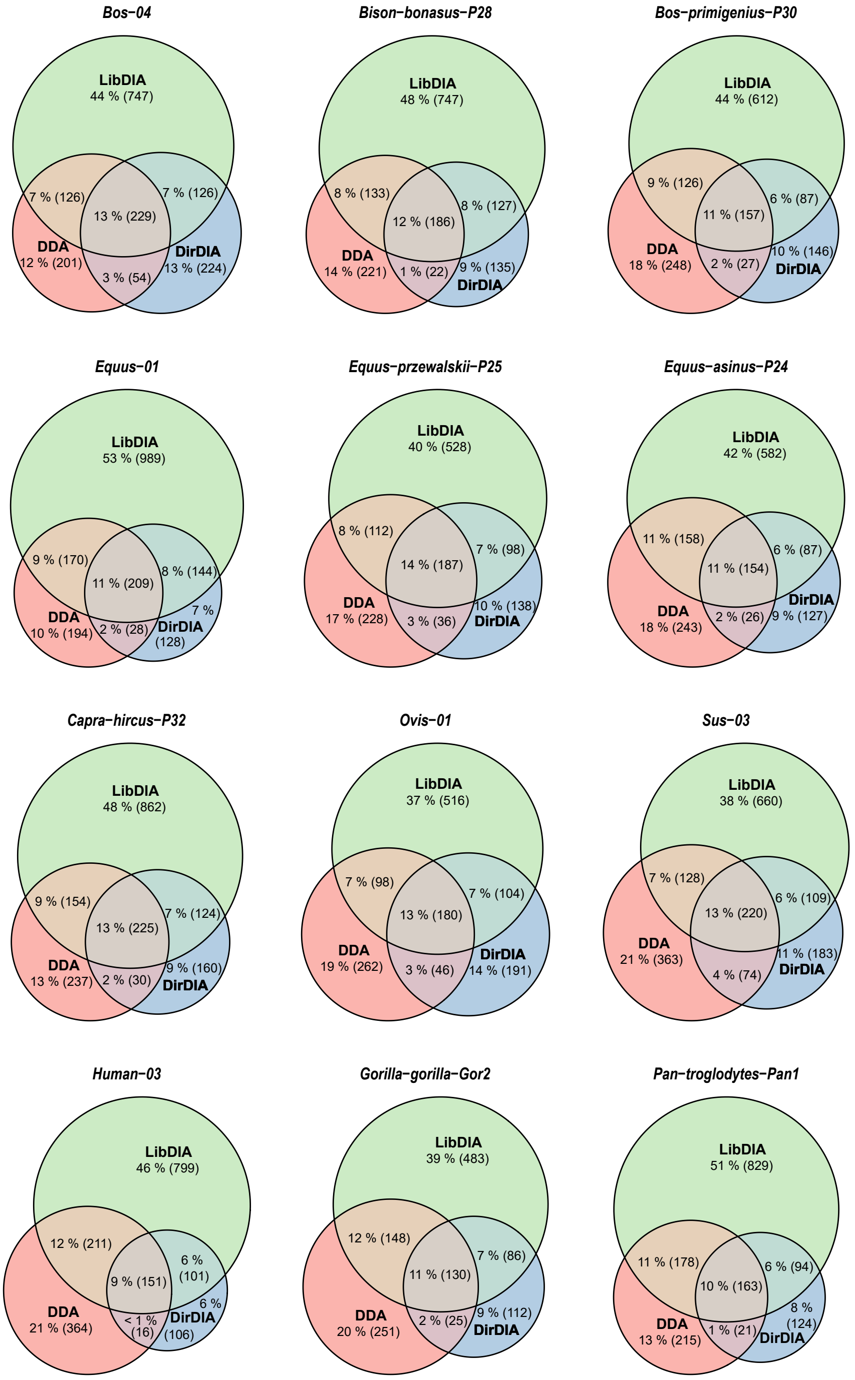

Fig. S12 | Peptide overlap between acquisition methods. Euler diagrams based on the semi-specific peptide identifications. Each diagram represents one randomly selected sample for each reference species indicating peptide identifications and overlaps obtained with library-based DIA, Direct DIA, and DDA. The area is proportional to the peptide count within each experiment. No scaling was done between the experiments. Percentages indicate relative number of peptides and the absolute numbers of peptide identifications are in parenthesis.

Supplementary Note 13: Gene-wise sequence coverage

Objectives

To investigate the stability and degradation of specific genes over time, we compared the absolute sequence coverage, i.e. number of identified amino acids, in the three sample sets of different archaeological age.

Results

Most of the covered sequences were concentrated in the two collagen type 1 chains and the number of identified amino acids decreased with higher sample age of the archaeological samples from Salpetermosen and Portugal (fig. S13). Some genes showed a stronger decrease of coverage with progressive protein diagenesis, such as chondroadherin and fetuin, which were almost completely absent in the Portuguese samples. Overall, the peptide retrieval was most stable over time for collagens.

Conclusions

Our data confirmed that collagens are a more persistent source of protein sequence information, when it comes to very old and degraded bones. As we showed in the main article, phylogenetic placement was still possible at lower non-collagenous sequence coverage (fig. 4, 5), albeit at lower resolution and robustness. On the other hand, collagen sequence identification was very stable and facilitated species identification over time periods of more than 50,000 years. It should be noted that the absolute sequence coverage may be a good approximation for the achievable species resolution, but it is skewed when it comes to collagens. Due to their highly conserved and partially redundant sequence, their informative power is lower than that of non-collagenous proteins. In general, any variation in sequence coverage will be taken into consideration by the site-based species identification workflow.

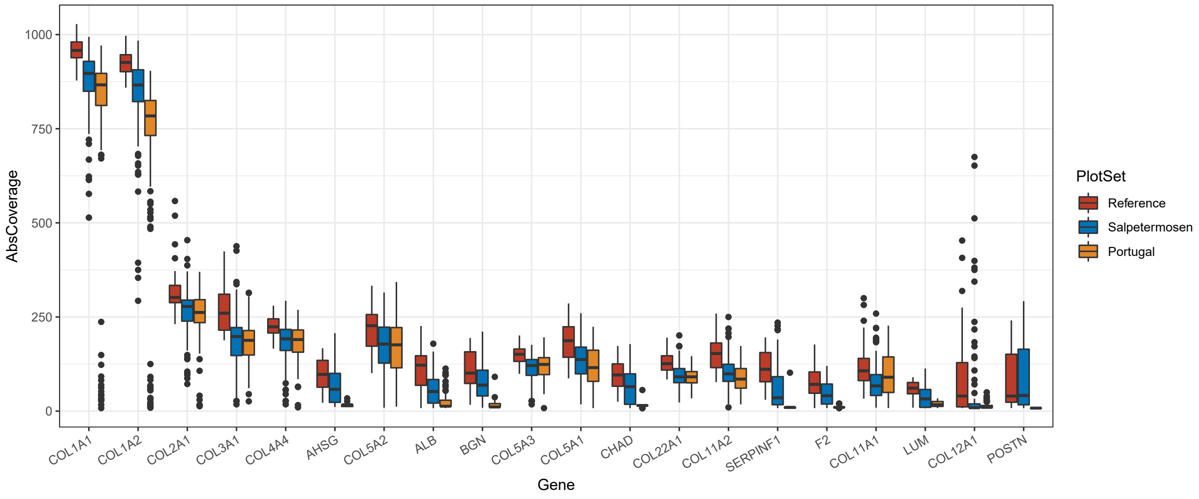

Fig. S13 | Absolute sequence coverage of the 20 genes included in the SPIN analysis by library-based DIA for the three sample sets. Boxplots include the median as a horizontal line, the lower and upper quartile as boarders of the box, and whiskers indicating 1.5 times the interquartile range. Datapoints outside of the whisker range are displayed individually. The fill color indicates the archaeological site. The medieval reference bone samples (n = 35 biologically independent samples) are marked in red, the Iron Age samples (n = 126 based on 63 biologically independent samples measured in 2 biological replicates) in blue, and the middle-late Palaeolithic samples (n = 213 biologically independent samples) in orange.

Supplementary Note 14: Protein deamidation quantified by SPIN

Objectives

Using deamidation as a marker for peptide authenticity [4] and protein preservation [5] has been actively debated in the field of palaeoproteomics. Independent of the debate, we wanted to find out, whether the short LC-MS/MS method and data-independent acquisition would be suited for quantifying deamidation of glutamine and asparagine residues and how much deamidation rates correlated with the sample preservation state. Therefore, we assessed the state of deamidation in a semi-quantitative manner by relative precursor counting, which we did by dividing the number of identified precursors with deamidated residues by the total number of glutamine- or asparagine-containing precursors. We were particularly interested in the reproducibility of the deamidation rates by the three different peptide identifica-

tion methods library-based DIA, DirectDIA, and DDA.

Results

The calculated deamidation rates correlated best with a pearson correlation of 0.962 between the two different DIA approaches (fig. S14), which was expected because both analyses were based on the same raw files. However, the Pearson correlations of the two DIA appraoches with the deamidation rates determined based on DDA were also higher than 0.9. The overall distribution of deamidation rates indicated the lowest rate of deamidated precursors in the Danish Salpetermosen sample set and the highest rate in the Portuguese samples.

Conclusions

We did not expect the good correlation of the deamidation rates between the three analytical aproaches, as the short gradients complicate baseline separation of unmodified peptides from their deamidated counterparts and the isotope envelopes are largely overlapping. Due to the consistent results obtained with all three analytical approaches, it is fair to assume that the SPIN workflow is suitable for semi-quantitative analysis of protein deamidation. Comparing the deamidation rates between the three sample sets, it seems counter-intuitive that the rate is lowest for the Iron-Age samples from Salpetermosen and not for the medieval reference bones. However, it has previously been described that deamidation does not only correlate with age but also depends heavily of the preservation conditions like moisture and oxygen levels [5]. The Portuguese samples, which were by far the oldest, had the highest deamidation rates. Possibly the most important conclusion based on these results is that PTM analysis is generally possible by SPIN and the data analysis can be adapted to cover other diagenesis-linked or biological protein modifications.

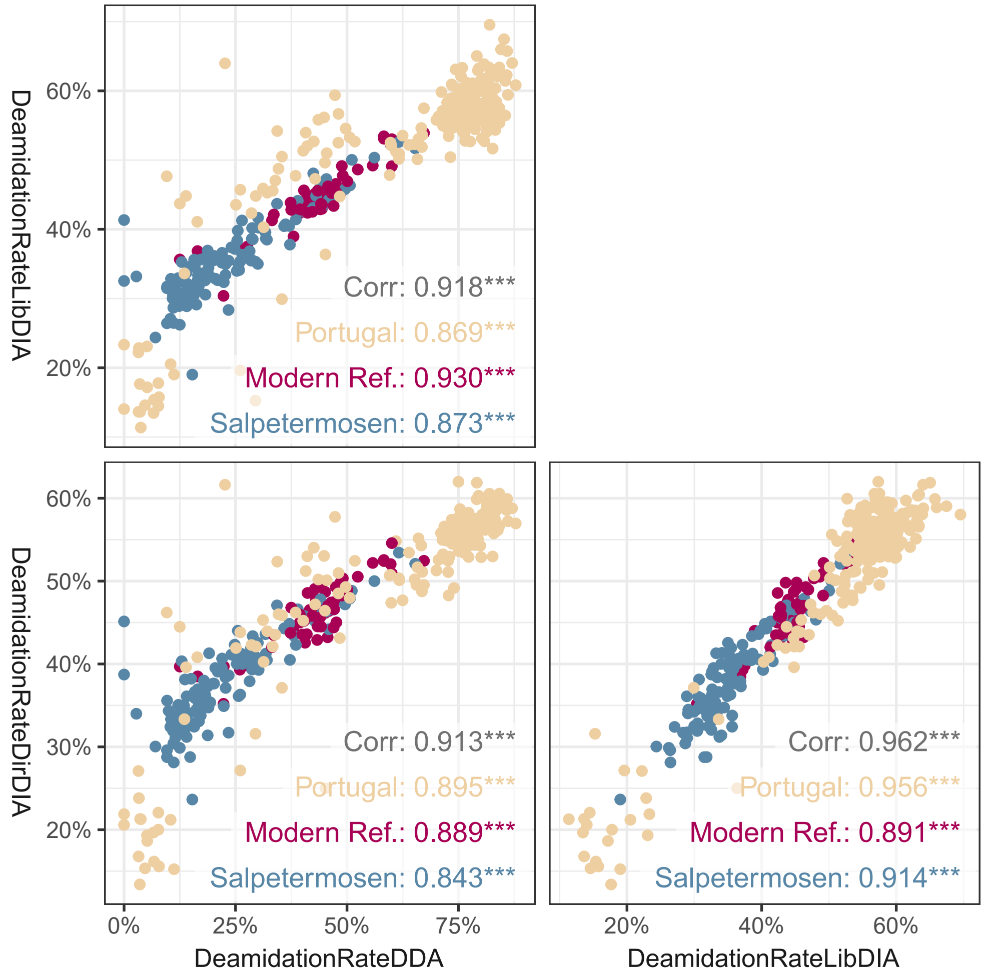

Fig. S14 | Deamidation rates calculated as relative spectral counts of deamidated and non-deamidated peptides containing an N or Q residue for all samples in the SPIN dataset. The medieval reference bone samples (n = 35 biologically independent samples) are marked in pink, the Iron Age samples (n = 126 based on 63 biologically independent samples measured in 2 biological replicates) in blue, and the middle-late Palaeolithic samples (n = 213 biologically independent samples) in sand color. Pairwise scatter plots are shown for the three different peptide identification methods. Numbers indicate Pearson correlations for the entire sample set in grey and in colored for the respective sample sets.

Supplementary Note 15: Annotation of DIA spectra - example of a potential great ape

Objectives

DIA analysis has not been commonly used in routine analysis for species identification. Due to the multiplexed MS2 scans with fragments from multiple precursors,

DIA data is more complex to interpret. Here, we tested whether this data type is suitable for manual validation of particularly interesting species-specific peptide identifications. We picked a peptide unique to great apes from one of the oldest samples in the SPIN study GdC-14, which was assigned to great apes by the species identification algorithm but was below the protein abundance QC threshold.

Results

The most straightforward tool for viewing annotated spectra was Spectronaut's included "Peptide data match" function. As it currently does not feature a usable export function, we only used it to identify solid precursor identifications, their retention time, and DIA window. With these information, we could find the respective MS2 scans in the raw data and enter them into the "Interactive Peptide Spectral Annotator" online tool [6] together with the peptide sequence and modifications. As an example, we looked at the peptide GEPGVVGAVGTAGPSGPSGLPGER that was only found in the middle Palaeolithic sample GdC-14 and in our great ape reference samples. The spectral annotation of the DIA scan revealed 12 matching y-ions and 9 matching b-ions along the peptide sequence besides fragments from one or more other precursors isolated in for the MS2 scan (fig. S15a). To check specificity for great apes, we used the peptide sequence for a BLASTp search [7] and displayed the search results as a phylogenetic tree (fig. S15b).

Conclusions

Spectra from DIA experiments could be used for validating peptide identifications by spectral annotation. Compared to validation of DDA spectra, there were more non-identified peaks from other precursors, which was expected due to the co-isolation of multiple precursors in DIA mode. We chose to visualize the precursor identification in a single MS2 scan because this is the established way of peptide validation in DDA data. However, DIA search algorithms internally do not use this perspective for scoring peptide identifications and instead extract ion traces for the precursor and fragment masses, which would be an alternative way of visualizating peptide identifi-

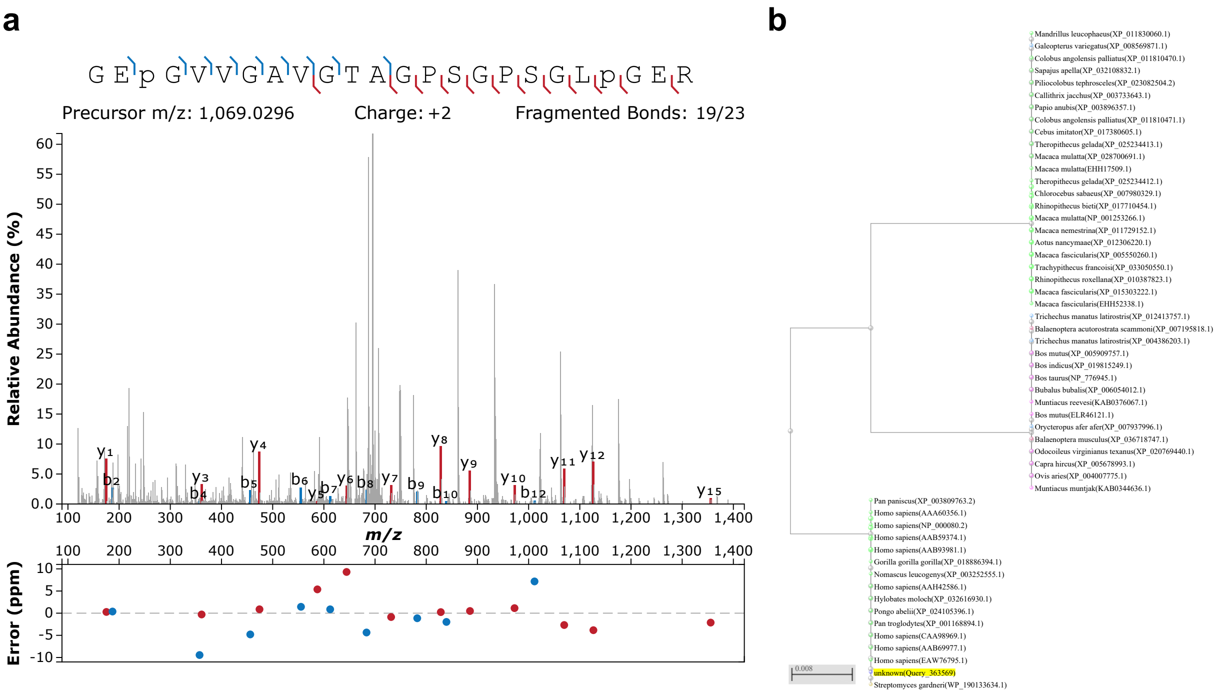

Fig. S15 | Spectral validation and phylogeny of peptide GEPGVVGAVGTAGPSG-PSGLPGER. The peptide was picked based on similarity between the putative human sample GdC-14 and the great ape reference samples. **a**, Annotation of the DIA MS2 scan at the apex of the peak by the "Interactive Peptide Spectral Annotator" [6]. The retention time and DIA window were extracted from Spectronaut. Only b- (marked in blue) and y-ions (marked in red) were annotated with up to 10 ppm mass accuracy. **b**, Sequence uniqueness to great apes was confirmed by BLASTp search. Results were visualized in the phylogentic tree view based on the searched peptide sequence.

cations.

Particularly for samples that were assigned to a particularly important species or had data quality below the quality control thresholds, manual validation was a viable option for confirming peptide identifications. Although much more reliable than MS1-based identification by peptide-mass-fingerprinting, the confidence could be increased by validation of a DDA scan, where all fragments can normally be assigned to the same precursor. In case of the example in figure S13, we were able to demonstrate that the peptide specific to great apes was confidently identified in sample GcD-14 by DIA analysis, which makes it likely that this bone belonged to a human or great ape.

Supplementary Note 16: Phylogeny of the complete SPIN database

Objectives

We reduced the protein database used for SPIN to only 20 genes across all available mammalian species, in order to facilitate multiple sequence alignments and manual validation of the aligned database. To ensure that the sequence information in these 20 genes is enough to reproduce the phylogeny of the included species, we created one consensus sequence per species and merged the 20 genes for creating a phylogenetic tree, as described in the main article.

Conclusions

The species grouping based on the protein database did resolve the included mammalian species phylogeny (fig. S16). Although even the placement of poorly annotated species with few protein sequences was correct in many cases, we set a minimum threshold of 15 annotated genes for a species to be included in the final SPIN analysis. This way, we were able to reduce artifacts based on very few potentially false peptide identifications in these databases. In the future, the database can be supplemented with new protein sequences, for instance by predicting them from high quality genomes.

Table S3 | Species re-assignment for middle to late Palaeolithic samples from Portugal predating human domestication of animals. In many cases, protein sequence databases only contain data for domesticated animals and not for their wild ancestors. Therefore, the most probable wild species were inferred based on morphological and genetic similarity of species.

SPIN species	Probable species
Bison bison;Bison bonasus;Bos indicus;Bos mutus;Bos primigenius;Bos taurus	Aurochs;Bison
Capra hircus	Ibex
Ovis aries	Mouflon
Equus asinus	Wild ass
Equus caballus;Equus przewalskii	Wild horse
Odocoileus virginianus	Deer
Oryctolagus cuniculus	Rabbit
Sus scrofa	Wild boar
Vulpes vulpes	Fox

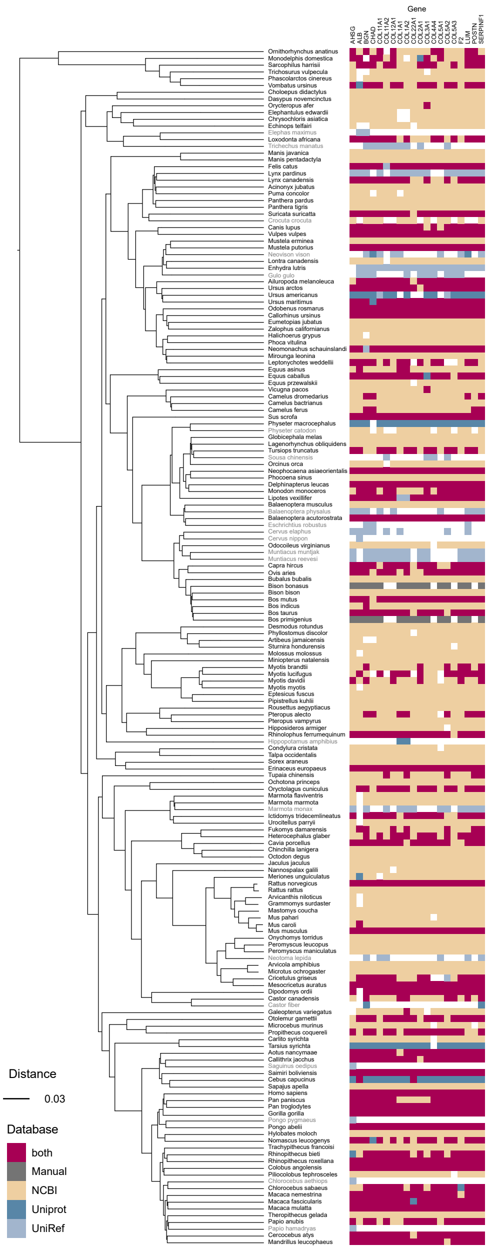

Fig. S16 | Complete database phylogeny. Sequences of the 20 most abundant genes in a bone proteome were sourced from Uniprot and NCBI to create the protein database used in SPIN. Taxa with less than 15 genes in the database were excluded in the course of the analysis and are indicated by grey font. Color indicates database source with dark blue for sequences with annotated gene name from Uniprot, light blue for Uniprot with gene name added from UniRef, sand color for NCBI, pink for sequences available in both databases, and grey for manually added sequences. A consensus sequence was created for each gene and species to create the phylogenetic tree using FastTree [8] in amino acid mode and displayed by FigTree.

Supplementary Note 17: Timings and throughput of SPIN analysis

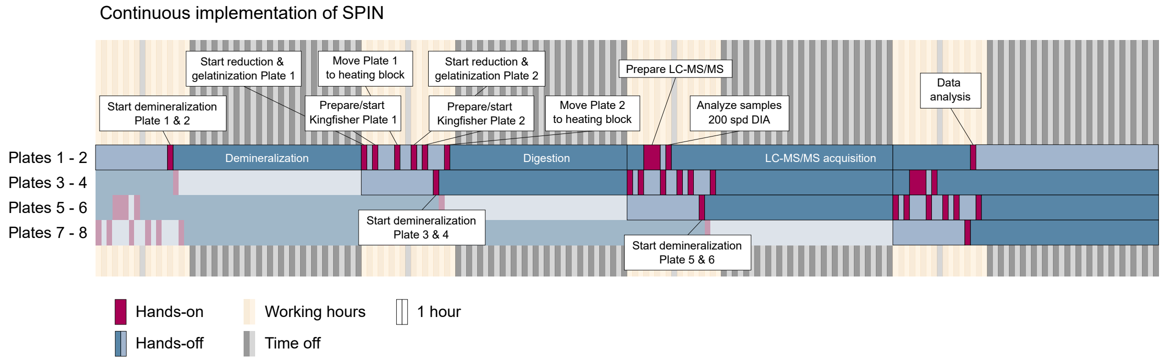

Fig. S17 | Example schedule of the steps needed for SPIN analysis when carried out by a single laboratory technician. Time required for each step was estimated based on subjective laboratory experience and will vary between operators. The scheme represents four working days with 4 times 8 working hours. Tasks are scheduled to allow for the parallel preparation of 8 x 96 samples, over the four days. The time of day is indicated by light sand color for working hours and in grey for resting hours. Hands-on steps are shown in pink, whereas breaks or incubation times are shown in blue.

Discussion

When parallelized, SPIN analysis can be operated at a continuous throughput of 200 samples per day by a single laboratory technician using a single Kingfisher robot and a single LC-MS/MS. Most hands-on steps indicated in Fig. S17 can be done in less than 30 min by a trained worker, leaving enough time for switching between the tasks. We confirmed the feasibility of this workflow by processing the 213 samples from Portugal in the same sequence but without parallelization. A continuous throughput of 200 samples per day from bone powder to sequence identification cannot be reached with conventional PMF analysis, particularly due to the manual data interpretation (several hours for 200 samples), MALDI target plate spotting (several hours per about 180 samples), and additional peptide elution after purification.

Supplementary References

[1] Hughes, C. S. *et al.* Ultrasensitive proteome analysis using paramagnetic bead technology. *Mol. Syst. Biol.* **10**, 757 (2014).

[2] Batth, T. S. *et al.* Protein aggregation capture on microparticles enables multipurpose proteomics sample preparation. *Mol. Cell. Proteomics* **18**, 1027–1035 (2019).

[3] Cleland, T. P. Solid digestion of demineralized bone as a method to access potentially insoluble proteins and Post-Translational modifications. *J. Proteome Res.* **17**, 536–542 (2018).

[4] Ramsøe, A. *et al.* DeamiDATE 1.0: Site-specific deamidation as a tool to assess authenticity of members of ancient proteomes. *J. Archaeol. Sci.* **115**, 105080 (2020).

[5] Schroeter, E. R. & Cleland, T. P. Glutamine deamidation: an indicator of antiquity, or preservational quality? (2016).

[6] Brademan, D. R., Riley, N. M., Kwiecien, N. W. & Coon, J. J. Interactive peptide spectral annotator: A versatile web-based tool for proteomic applications. *Mol. Cell. Proteomics* **18**, S193–S201 (2019).

[7] Altschul, S. F., Gish, W., Miller, W., Myers, E. W. & Lipman, D. J. Basic local alignment search tool. *J. Mol. Biol.* **215**, 403–410 (1990).

[8] Price, M. N., Dehal, P. S. & Arkin, A. P. FastTree 2–approximately maximum-likelihood trees for large alignments. *PLoS One* **5**, e9490 (2010).
