# Supplementary material for: SPIN enables high throughput species identification of archaeological bone by proteomics
Source: Nat Commun. 2022 May 5;13:2458. doi: 10.1038/s41467-022-30097-x (PMC9072323; doi:10.1038/s41467-022-30097-x)
Supplement: Supplementary file 3 — Description of Additional Supplementary Files [file 41467_2022_30097_MOESM3_ESM.pdf]

Description of supplementary data

This README describes the supplementary data associated with the publication "SPIN - Species by Proteome INvestigation" by Rüther et al. 2022

Supplementary Data 1: Gene ranking by sequence coverage

Identified genes ranked by decreasing peptide spectrum matches in the 4 spectral libraries for cow (Bos taurus), horse (Equus caballus), sheep (Ovis aries), and pig (Sus scrofa). The top 20 genes account for about 99 % of PSMs and were therefore selected for constructing the aligned species database used for SPIN.

Supplementary Data 2: SPIN species identifications

Sheet 1 contains the results obtained by merging the library-DIA and DirectDIA results by adapting the Direct DIA species for every sample with an identification that was not part of the spectral libraries. Sheet 2 contains DDA-based species identifications, and sheets 3 and 4 contain individual species results from DirectDIA or library-DIA data. The species results can be mapped to the more detailed metadata in table S2 using the "Raw file" or "R.FileName" columns. The final species output after fine grouping, if applicable, and quality control can be found in the "Species" column. In case of multiple possibilities, all equally likely species names are specified in alphabetical order separated by semicolons. Samples below the relative protease intensity thresholds are marked as "signal too low" and samples that did not pass the target-decoy based FDR are marked with "below FDR", in the "Species"column. The "RoughSpecies" column indicates the raw output of the site-based species inference algorithm, while the "FineSpecies" column shows the most likely species after fine grouping using species marker peptides. The "SiteCount" column contains the number of sites, i.e. the absolute number of identified amino acids, matching the identified species. "RelProtInt" indicates the relative protease intensity.

Supplementary Data 3: SPIN-derived consensus sequences

Consensus sequences for each sample in the SPIN study based on all peptides identified by library-based DIA ("LibDIACoverage" column), DirectDIA ("DirDIACoverage" column), or DDA ("DDACoverage" column). The consensus amino acid at each site was selected by the highest number of precursor identifications.

Supplementary Data 4: Sample metadata

Sample metadata for all samples used in the SPIN study. The data can be mapped to the species results in the SPIN study using the "Raw file" or "R.FileName" columns. For bones used as species references, the species is provided in the "RefSpecies" column. For the bones from the Salpetermosen site in Denmark that were analyzed morphologically, the morphology-based species group and most likely species are listed in the "MorphologyGroup (Species)" column. Museum collection or archaeological excavation IDs are provided in the "Identifier" column. If applicable, the archaeological site location and dating information are provided in the "Location", "Time period", and "Age" columns.

Supplementary Data 5: Comparison results of SPIN vs. MALDI-TOF

Species results of the comparison between SPIN and PMF-based ZooMS (Fig. 6). Identical peptide extracts from all 46 reference samples, 20 representative samples from Salpetermosen, and 21 representative samples from Portugal were analyzed by LC-MS/MS and by MALDI-TOF MS. SPIN data was analyzed with the automated data interpretation algorithm, whereas the ZooMS data was interpreted manually. The species output was translated for better comparability and simpler plotting. Original species output and simplified plot labels are listed in the table.
