# Supplementary material for: SPIN enables high throughput species identification of archaeological bone by proteomics
Source: Nat Commun. 2022 May 5;13:2458. doi: 10.1038/s41467-022-30097-x (PMC9072323; doi:10.1038/s41467-022-30097-x)
Supplement: Supplementary file 5 — Reporting Summary [file 41467_2022_30097_MOESM5_ESM.pdf]

nature research

Corresponding author(s): Jesper Velgaard Olsen

Last updated by author(s): Dec 16, 2021

Reporting Summary

Nature Research wishes to improve the reproducibility of the work that we publish. This form provides structure for consistency and transparency in reporting. For further information on Nature Research policies, see our Editorial Policies and the Editorial Policy Checklist.

Statistics

For all statistical analyses, confirm that the following items are present in the figure legend, table legend, main text, or Methods section.

n/a	Confirmed	
☐	☒	The exact sample size (n) for each experimental group/condition, given as a discrete number and unit of measurement
☐	☒	A statement on whether measurements were taken from distinct samples or whether the same sample was measured repeatedly
☒	☐	The statistical test(s) used AND whether they are one- or two-sided<br>*Only common tests should be described solely by name; describe more complex techniques in the Methods section.*
☒	☐	A description of all covariates tested
☒	☐	A description of any assumptions or corrections, such as tests of normality and adjustment for multiple comparisons
☐	☒	A full description of the statistical parameters including central tendency (e.g. means) or other basic estimates (e.g. regression coefficient) AND variation (e.g. standard deviation) or associated estimates of uncertainty (e.g. confidence intervals)
☒	☐	For null hypothesis testing, the test statistic (e.g. F, t, r) with confidence intervals, effect sizes, degrees of freedom and P value noted<br>*Give P values as exact values whenever suitable.*
☒	☐	For Bayesian analysis, information on the choice of priors and Markov chain Monte Carlo settings
☐	☒	For hierarchical and complex designs, identification of the appropriate level for tests and full reporting of outcomes
☐	☒	Estimates of effect sizes (e.g. Cohen's d, Pearson's r), indicating how they were calculated

Our web collection on statistics for biologists contains articles on many of the points above.

Software and code

Policy information about availability of computer code

Data collection	Xcalibur 3.1-4, Chronos 4.9.2.0, Evosep plugin 1.4.81.0, Chromeleon 6.8
Data analysis	MaxQuant 1.6.0.17, Spectronaut 14.5.200813 and 14.11.210528, R studio 1.3.1093, Inkscape 1.0.1, MUSCLE 3.8.425, UGENE 37, AliView 1.26, FastTree 2.1.11, FigTree 1.4.4<br>R-packages: base R 4.0.3, bit64 4.0.5, data.table 1.13.4, eulerr 6.1.0, future 1.21.0, future.apply 1.6.0, ggplot2 3.3.2, ggrepel 0.9.1, ggsci 2.9, gmp 0.6-1, progressr 0.7.0, stringi 1.5.3, stringr 1.4.0, RColorBrewer 1.1-2, scales 1.1.1<br>Scripts in R written for usage in RStudio and the sequence-aligned protein database are publicly available under Creative Commons Attribution 4.0 International license on Zenodo (DOI: 10.5281/zenodo.6406044).

For manuscripts utilizing custom algorithms or software that are central to the research but not yet described in published literature, software must be made available to editors and reviewers. We strongly encourage code deposition in a community repository (e.g. GitHub). See the Nature Research guidelines for submitting code & software for further information.

Data

Policy information about availability of data

All manuscripts must include a data availability statement. This statement should provide the following information, where applicable:

- Accession codes, unique identifiers, or web links for publicly available datasets
- A list of figures that have associated raw data
- A description of any restrictions on data availability

The mass spectrometry proteomics data have been deposited to the ProteomeXchange Consortium via the PRIDE(Perez-Riverol et al. 2019) partner repository with the dataset identifier PXD024487 [Reviewer access: reviewer_pxd024487@ebi.ac.uk Password: tjMnzCQ2]. Protein sequence databases were sourced from Uniprot Knowledgebase release 2020_06 and NCBI RefSeq release 201 (July 2020).

Field-specific reporting

Please select the one below that is the best fit for your research. If you are not sure, read the appropriate sections before making your selection.

- [x] Life sciences
- [ ] Behavioural & social sciences
- [ ] Ecological, evolutionary & environmental sciences

For a reference copy of the document with all sections, see nature.com/documents/nr-reporting-summary-flat.pdf

Life sciences study design

All studies must disclose on these points even when the disclosure is negative.

Sample size	410 bone specimens. All details for individual samples are provided in Supplementary table S2. All bones were sampled with consent by museum conservators and archaeologists.
Data exclusions	All data were included.
Replication	Biologically independent replicates were measured for known reference bones between 3 and 10 replicates. Technical injection duplicates were measured for the Salpetermosen dataset. Replication success varied with sample types and data analysis strategy and can be found in the manuscript and supplementary data 2. All method optimization experiments were prepared as workflow triplicates and results were displayed as mean and standard deviation (supplementary material).
Randomization	Mass spectrometric acquisition order was randomized including laboratory and injection blanks. Methods optimization samples were measured in random order.
Blinding	Blinding was carried out for the species determination for Salpetermosen dataset to keep morphological and protein sequence based species analysis separate.

Reporting for specific materials, systems and methods

We require information from authors about some types of materials, experimental systems and methods used in many studies. Here, indicate whether each material, system or method listed is relevant to your study. If you are not sure if a list item applies to your research, read the appropriate section before selecting a response.

Materials & experimental systems

n/a	Involved in the study	
x		Antibodies
x		Eukaryotic cell lines
	x	Palaeontology and archaeology
x		Animals and other organisms
x		Human research participants
x		Clinical data
x		Dual use research of concern

Methods

n/a	Involved in the study	
x		ChIP-seq
x		Flow cytometry
x		MRI-based neuroimaging

Palaeontology and Archaeology

Specimen provenance	Sample owners are included as co-authors on the manuscript. Samples from National History Museum of Denmark have been collected with authorization (100720#8741). M.M. Benedetti, J. Haws, and N. Bicho approved the export of the samples from Portugal.P. Pantmann and P. Bangsgaard approved the sampling of bone fragments from the Salpetermosen site in Denmark.
Specimen deposition	Remaining unused samples have been returned to the owners.
Dating methods	No dating has been done as part of this manuscript. Listed radiocarbon dates were retrieved from published data.

- [ ] Tick this box to confirm that the raw and calibrated dates are available in the paper or in Supplementary Information.

Ethics oversight	No ethical approval was required because study objects were disposable bone fragments and sampling was reduced to less than 100 mg representing a small fraction of the bones.

Note that full information on the approval of the study protocol must also be provided in the manuscript.
